# A Circular Economy: Where Will It Take Us?

**DOI:** 10.1007/s43615-021-00013-4

**Published:** 2021-03-09

**Authors:** Jim Hart, Francesco Pomponi

**Affiliations:** 1 Resource Efficient Built Environment Lab (REBEL), Edinburgh Napier University, 10 Colinton Road, Edinburgh, EH10 5DT UK; 2 Cambridge Institute for Sustainability Leadership (CISL), University of Cambridge, 1 Trumpington Street, Cambridge, CB2 1PZ UK

**Keywords:** Circular business models, Sharing economy, Circular economy, Sustainability, Technology

## Abstract

The avalanche of environmental challenges, from local to global and back, has prompted responses at all levels from personal to inter-governmental. The results of these responses have fallen in the range between useful and counterproductive, with many examples on each side, but the scale of the overall challenge continues to escalate. Moving towards a zero-carbon global economy through absolute reductions in fossil fuel usage is a sure way of mitigating climate change, and a range of environmental, social and economic benefits would follow. The case for a Circular Economy (CE), however, is less clear. Whilst some CE initiatives may lead to the decoupling of economic growth from resource extraction, this does not necessarily equate to reducing the rate of extraction. Thus, the contribution of CE to the achievement of environmental objectives globally cannot be taken for granted. In terms of social impact, the best that can be said is that CE might be neutral. Technologies that promote the ‘sharing economy’ for instance, often suggested as a crucial CE strategy, create opportunities for individual wealth accumulation, but are also a route to the gig economy and the casualisation of labour. CE is arguably a business imperative, but definitive evidence to support the idea of a circular economy that meets social and environmental goals needs development.

## Introduction

Only a particularly uncurious person could be unaware of the modern scourges of the natural environment, and their increasing incursions into our lived experiences. Familiar examples, amongst many, include climate change [1], air pollution [2]; soil erosion [3] and deforestation [4]. The number of environmental challenges mounts up, with terms such as micro-plastics [5], and insectageddon [6] entering the lexicon in response to new information and data. The scale of individual challenges also continues to increase as emissions targets are missed and the increasing concentration of greenhouse gases (GHG) in the atmosphere continues to accelerate [7].

Whilst such challenges are sometimes tackled as discrete problems, their inter-relationships indicate the need to tackle the root causes. In response to the need for fundamental change in the way we produce and consume, or the need to be seen to be responding to these challenges, the concept of a circular economy (CE) has recently gained traction. This offers an alternative to the more familiar linear ‘make-use-dispose’ model of production and consumption.

This article offers a perspective on potential implications of CE implementation in terms of sustainability, considering possible benefits and costs to the environment, society and the economy, globally. Whilst not a systematic review of the literature, the authors use a selection of relevant and recent literature and examples to highlight areas where greater understanding is needed of how CE can be harnessed to humanity’s collective benefit. It is also a plea for participants in the CE to evaluate their contribution to wider objectives, and not use involvement in CE as a ‘get out of jail free’ card.

The article begins with a discussion of circular economy in the context of policy and sustainability (‘Policy Response’ section) before looking at the role technology might have in finding a way forward for CE and discusses the absence of social benefit in some visions (‘Technology to the Rescue?’ section). This leads on to a discussion of steering CE away from an ‘economy first’ (and everything else last) in the ‘Circular Economy—Economy First?’ section, and ending with a look into the future.

## Policy Response

The possible responses to environmental challenges are as numerous and varied as the problems themselves. Positive responses can range from the individual micro-effort to global agreements to force change. Citizens are politely encouraged to ‘do their bit’ by turning thermostats down and eschewing single-use plastic shopping bags, but such bottom-up efforts are unlikely to yield change at the scale required when ‘the system’ still depends on a paradigm of continuous growth which relies on selling more stuff to an ever growing number of citizens [8]. At the other end of the scale, governments can work together—as they have with the Montreal Protocol, and the Paris Agreement for instance, and set and enforce global caps on certain damaging activities with global effect.

In between these two extremes, there exists a huge range of possible responses from communities, entrepreneurs, corporations, and governments. These can, for instance, involve the development of new technologies and products, the accelerated roll-out of well-proven (but perhaps expensive) technologies, sustainable business models, and any number of types of incentives and regulatory measures. Arguably, underpinning such measures with a coherent philosophy and framework can help to mobilise society and ensure widespread engagement. This is the role of the umbrella terms ‘sustainability’ and ‘circular economy’.

### Sustainability and Circular Economy

Sustainability and CE mean different things to different people, and—indeed—may be regarded as ‘contested concepts’, as noted by Korhonen et al. [9] in connection with CE. However, most reasonable observers would recognise that both should offer the prospect of progress towards environmental goals, amongst other things. The concept of sustainability [10] generally requires that attention is spread across environment, society, and economy, and their inter-connections. This is a rebuke to hard-nosed capitalists who believe in profit and economic growth at all costs. Equally, it is a reality check for environmentalists who want to put nature first, and everything else behind. There is little wrong with the concept of sustainability, other than the significant issue of its failure to be sufficiently understood and embraced [11, 12]: the continuing pre-eminence of economic growth metrics in public discourse is evidence of this [13, 14].

The alternative (or, potentially, complementary) vision of a CE has been put forward, and endorsed by organisations and businesses around the world, especially in the European Union (EU) [15] and China [16]. The sheer number of available definitions of CE is frequently commented on [17], and it may be too late for a unifying definition to emerge, as the concept has emerged through cross-breeding of related schools of thought over recent decades (many highlighted by Borrello et al. [18]) and undergone mutations to adapt to selection pressures imposed by different stakeholders, understanding the issue from different perspectives (e.g. macro, meso and micro scales [19]). The best that can be hoped for is that definitions with a clear pro-environment agenda offer sufficient gravitational pull to counter those primarily biased towards economic growth. Definitions sometimes include abstract concepts that may absorb more light than they shed [20]: for instance ‘an economy that is restorative and regenerative by design’ [21], (how and what does an economy regenerate, and who will design it? Morseletto’s review [22] finds potential meaning in ‘restorative’ in the context of ecological improvement, but less in ‘regenerative’). Some present CE as an economic strategy with environmental benefits on the side; and others suggest it is primarily a restatement of the waste hierarchy (reduce, reuse, recycle), although some implementations of CE emphasise ‘recycle’ more than ‘reduce’, thereby subverting it to the cause of unsustainable business as usual. Make do and mend [23] was tried during the war, but since then the idea and practice of recycling and recovery are the only alternative strategies to gain real traction [24], and deep CE-thinking is needed to push beyond this [25, 26]. Haas et al. [27] make the point that the ultimate objective of a sustainable CE should be absolute global reductions in resource extraction, waste and emissions. The success or failure of ‘the’ CE can be thus determined; on the other hand, CE-focussed projects, products and policies have to be judged on the much less clear measure of whether they demonstrably contribute to the greater goal.

A CE is not just about how we make and dispose of objects: how we use them, how we share them, who owns them, and how they are maintained and paid for are all grist to the CE mill, covered by the term Circular Business Models (CBMs) [28]. Altogether, this is too much to communicate within a pithy definition, and newcomers to CE are invited to consider it in relation to things as they are—styled in CE literature as the ‘linear economy’ with its make-use-dispose approach. A circular economy appears to offer something for everyone. If the linear economy leaves you in a state of despair because of its wasteful treatment of natural resources, then you may warm to CE. On the other hand, a businessperson comfortable with the *status quo* will require a sense of opportunity to excite their interest: this is often implied by suggestions of lower costs and higher margins [28, 29]. And those with heightened senses will hear a bandwagon starting to roll and catch a whiff of opportunity from government support (innovation funding and soft loans, for instance), and investment and custom from other businesses also wanting to get on board. Strategic partners of the Ellen MacArthur Foundation (EMF)—a well-known advocate of CE—include global names in technology, fashion, automotive, electrical, financial services, food, and household products. Whilst there are indications of worthwhile initiatives, there is little sign that fundamental business models and resulting environmental impacts are changed.

### Cautionary Tales

In many cases, interventions on environmental challenges have yielded good results: governmental and inter-governmental work has resulted in huge progress on issues such as the stratospheric ozone hole [30], acid rain [31], and vehicular lead emissions [32]. Although not inspired by CE *per se*, these cases are beacons, showing that it is possible to pick off environmental challenges individually, and hinting at the potential power of systemic change, through CE, addressing problems collectively. However, in other cases, pro-environment interventions may have been counterproductive, in that purportedly green or circular policies have had substantial side effects [33]. One example from many is the promotion of wood as an ecologically sound choice of heating fuel: this has resulted in 7.5% of homes in the UK burning wood for heating [34], emitting air pollution in the form of NOx and particulate matter, even in urban areas where illegal levels of air pollution are frequently recorded [35]. Subsidies supporting the combustion of biomass are sometimes so poorly designed that the rewards for consumption of fuel have exceeded the cost, leading to excessive fuel consumption [36]. And the subsidies for renewable power in the UK have resulted in annual imports of 6 Mt of wood pellets per year from outside of the EU (95% of this from across the Atlantic), primarily for a single power station [37]. The carbon footprint associated with the processing and transport of this material is troubling enough, but to ignore the long wait for the carbon emitted at combustion to be re-sequestered by replacement forest is risky [38]. Turning to cropland, two-thirds of cropland required for the EU’s non-food requirements is from regions outside of the EU, and monitoring of displacement effects is needed [39]. Perhaps, as some suggest, the bioeconomy (compliant with many visions of CE) is the inevitable contemporary synergy between capitalism and biotechnology [40].

A case which resonates with the general population is single-use plastics leading to the pollution of our oceans and rivers with single-use plastics [41]. Following China’s ban on importation of plastic waste, parts of the waste industry elsewhere have found other outlets, with countries in South-East Asia experiencing increases in imports of hundreds of percent [42], vastly outstripping their capacity to manage the material in an environmentally responsible manner [43]. Bans and pricing mechanisms targeting plastic carrier bag consumption have been introduced around the world. In a sense, this looks like strong circularity [20], as it problematises the inflow of materials (whereas weak circularity focuses on outflows), but the collective impact is difficult to assess. Successes in some cases are counterbalanced by rebounds and negative side-effects in others [44]. These include increased demand for plastic garbage bags, a switch to more resource-intensive bags, and the substitution of banned single-use plastic bags with much heavier ‘bags for life’, which, despite their name are often discarded after one use because the small charge for such a bag is no disincentive when the single-use option is unavailable [45]. This might be corrected, for instance, by increasing the charges [46], including the differential between multi-use and single-use bags to reflect their different weights.

In these examples, we see that narrow focus on one aspect of an environmental problem can result in unwanted side effects. A genuinely holistic approach is required for such problem-solving, and merely invoking CE is not sufficient. It is unlikely that everybody involved in such policy development is unaware of the potential problems, but may have been overwhelmed by pragmatism and politics, whereby policies under development are trimmed in various ways to make them more palatable [12, 47–50]. Enforcement is replaced with voluntary commitment; fees and charges are set at low levels so as not to cause alarm; and over-the-top subsidy might be seen as a simple method for meeting a particularly challenging objective.

Summing up, in order to deliver genuine environmental benefits whilst minimising side effects, CE policy needs to be reinforced with a robust framework of impact assessment, packaged with measurability and enforceability.

## Technology to the Rescue?

It can be argued that new technologies offer an escape from the make-use-dispose treadmill that has increasingly caused environmental problems to mount up since the industrial revolution [51, 52]. The role of digital technologies in reducing demand for newsprint is a case in point [53]. On the other hand, technology also contributes to problems that CE seeks to solve: the energy demands of big data [54], and the material resources required to support planned obsolescence [55] are examples. Technology itself is neutral: it is the application that matters.

So how might technology be used to insert delays and loops into linear systems, thereby slowing the extraction and disposal of primary resources?

New materials and industrial processes offer one route to more circular products. In the popular imagination, 3D Printing is almost the epitome of modern manufacturing, being high-tech, accessible, and capable of manufacturing small runs of bespoke designs with minimal set-up. Whilst 3D Printing enables the manufacture of a wide range of products—from plastic gizmos to bridges and buildings—potentially cutting out waste from the manufacturing process, there is no guarantee that this will increase circularity overall [56].

An ever-increasing number of products are now available made from recyclate [57], and from industrial and agricultural waste streams, which is to be welcomed. However, such advances can entrench recycling and therefore business as usual: clothing lines that rely on a limitless stream of PET (polyethylene terephthalate) bottles are potentially an example [58], whilst also likely adding to the burden of marine microplastics [5]. But some agricultural and food industry wastes are an inevitable part of the food system essential to human civilisation, and if they can be harnessed to make the products we as a society actually need, then this is good news. But this only works if the waste infrastructure is developed in parallel with product development [59–61]. For instance, the benefits of compostable biopolymers, whereby nutrients return to the soil from whence they came, are only realisable if the materials are actually sent for composting. This requires either that all plastics are compostable, or that technology is up to the task of identifying and separating compostable polymers from the rest. The alternative is that compostable materials contaminate the plastics recycling stream, and are either incinerated or landfilled along with it. Whatever the ultimate objective, before we achieve bioplastics waste treatment nirvana, a messy and potentially long transition takes place [62].

### Technology and Society—the Sharing Economy and Circular Business Models

Technology is also a vital facilitator of the ‘sharing economy’ [63] (or ‘collaborative consumption’ [64] and ‘peer to peer economy’), which is seen by some as a pillar of the circular economy [65]. But the circular embrace of the sharing is not necessarily reciprocated, with—for instance—a report on the sharing economy not referring to the circular economy [66]. Digital platforms enable the sharing economy (or, according to Belk [67], ‘pseudo-sharing’ when commercial activity is dressed up as community asset sharing) by allowing users to increase the utilisation of their assets, which can be a positive outcome for all parties directly involved (the lender, the borrower, and the platform). However, the environmental and social benefits outweighing the costs is by no means a given. Even in the case of a transaction as apparently benign as sharing an electric drill through a CE-inspired tool library, one should ask whether the counterfactual would have been the purchase of an additional drill by the frustrated borrower or whether it would instead have been avoidance of the purchase of the plasterboard or shelving that was behind the supposed need for a drill. Give someone a fish and you feed them for a day; lend them a bottom trawler and you wipe out life on the seafloor and impact biodiversity and fish stocks for years to come.

Anything that breaks the cycle of demand stimulation, de-unionisation, centralisation of wealth, purchase, regret and disposal that typifies the linear economy is likely to be a move in the right direction [68], and CBMs are proposed as a solution. But evidence that they can leverage progress on all of these issues is lacking, and even the premise that if we aim to own less stuff, then less stuff will be made is debatable. Uber increases the utilisation of vehicles for instance, but has resulted in more, not fewer, cars being on the road [69]. In future, when a fleet of driverless app-based taxis replaces a much larger fleet of privately owned vehicles, environmental efficiencies will result, as such vehicles will likely travel further in their lifetimes than typical private vehicles, thereby delivering greater service per unit of resource. But there may be more traffic overall on account of the low costs per mile. And more again, because the low cost of the system will undermine existing public transport, it might in effect become the new public transport. Other sharing opportunities are also likely to indirectly promote car usage: for instance apps that promote the sharing of parking spaces [66] which, in reducing costs to the driver, increase accessibility of city centres to the private car, undermining local authority traffic management and pollution control strategies and potentially depriving them of income (ideally for the public good) from their own facilities.

Peer-to-peer apps are also promoting CBMs in the rag trade, aiming to slow the turnover of fast fashion. The income gained by the dedicated followers of fashion renting out their spare clothes will, however, enable them to invest in more spare clothes, whilst adding to the procession of delivery vehicle traffic. The fundamental problem is that platforms designed to promote the sharing of excess stuff can lead to the purchase of excess stuff in order to ‘share’ it, thereby growing an income: there is a fluid boundary between altruistic motive of sharing and the profit motive [70]. Just as a housing is now viewed—by owners—as an investment as much as somewhere to live, clothing will become a ‘gig’ or ‘side-hustle’, rather than stuff to wear. Instagram micro-celebrities have been suggested as enablers to a wider adoption of clothes-renting [71], and it is easy to imagine how this triggers in turn another loop of a new post each day, with a new purchase to show. A more circular fashion choice would be along the lines of buying fewer items, of higher quality, sustainably sourced, and repairable. Undoubtedly, environmental impact would be reduced, but so would profit.

Airbnb is a globally known case identifiable as a CBM as it enables the extraction of greater financial value from fixed resources (in this case, property), and in principle it could enable greater utilisation of spare rooms. The reality, though, is a distortion of the property market as investors build and buy in geographical hot-spots, hollowing out local communities and pouring more petrol onto the inferno of mass tourism [72, 73], whilst also undermining existing jobs in the hospitality industry [74, 75]: social and environmental sustainability are nowhere to be seen. Some of these risks are acknowledged by the Organisation for Economic Cooperation and Development [76]. Other business models in which the end-user is not the owner are also presented as CBMs, and described in such terms as ‘servitisation’ or ‘product as service’ models, but servitisation only contributes to environmental sustainability in quite narrow terms [77] and a possible consequence (arguably, indeed, the purpose) of servitisation is that the products become more accessible to users and are ultimately manufactured in greater quantity.

More behavioural science is needed to underpin claims of environmental sustainability, to avoid more examples of technologies intended to mitigate environmental impacts proving counterproductive to society [78]. Taking a broader perspective, CBMs may be environmentally meaningless in an otherwise linear economy.

### Asset Management

If technology’s involvement in the sharing economy does not necessarily benefit the environment, then where else can we look for examples of technology as an enabler of the circular economy? As so much of CE is about the utilisation and fate of ‘stuff’, then it is important that we know where our stuff is, how much of it there is, and what condition it is in. ‘Smart-circular product-service systems’ can capitalise on the increasing connectivity of all sorts of objects to enable remote monitoring and actuating of products, whilst offering analytical functions and growing product lifetime databases [79]. An EMF report [80] gives the example of prolonging vehicle lifetimes by using sensors to monitor usage and operating conditions, and alerting the owner to any abnormalities. In the construction of buildings, the same applies to all mechanical and electrical equipment: Building Information Modelling (BIM) can be used to house data on all aspects of the building [81], including materials passports, enabling the concept of ‘Building As Material Banks’ (BAMB) [82]. This allows rich data concerning the products and materials incorporated in a building to be passed to subsequent owners, facilitating repair and maintenance and—at the end of the building’s life—reuse.

Collaboration and design tools in the construction value chain, such as BIM, are enablers of CE in construction [83], but a deeper commitment to digital approaches by the construction industry will be required to maximise the potential benefits of BAMB [84]. Materials passports will need to be kept alive as buildings are maintained and modified over a period of decades, supported by—for instance—blockchain. Integrating BIM data on materials within a building with geographic information systems will enable the production of regional data and forecasts on material flows, allowing strategic planning regarding reuse of materials as buildings reach the end of life. It is difficult to find optimal circularity strategies for the design and operation of buildings in general: materials are stored in the fabric of buildings for such long, and indeterminate periods, and buildings may have to go through unforeseeable sequences of changes of use, ownership, repair and adaptation [83]. However, approaches suggested here can support several of the strategies identified by Eberhardt et al. [85], such as reuse of components and materials, optimising built form, and design for disassembly and adaptability. Regardless of whether or not CE is the motivating force, it is clear that technologies under the CE ‘umbrella’ have the potential to further extend the effective and efficient operating period of a range of products, delivering the benefits associated with resource efficiency.

## Circular Economy—Economy First?

Is the true purpose of CE economic growth rather than the amelioration of environmental pressures? One of the promises of CE is that it will result in the decoupling of economic growth from resource use but this is wishful thinking in the case of weak CE. The idea of decoupling itself is often poorly understood. In a study on low carbon cities in China [86 , p. 15] the authors report the successful achievement of such decoupling because: ‘the relationship between carbon emissions and economic development showed that GDP increased, and carbon emissions also increased; however, the economic growth rate was higher than the growth rate of carbon emissions’. This simply shows a reduced impact intensity per unit of GDP not that economic growth is free from additional environmental impact.

**Fig. 1:**
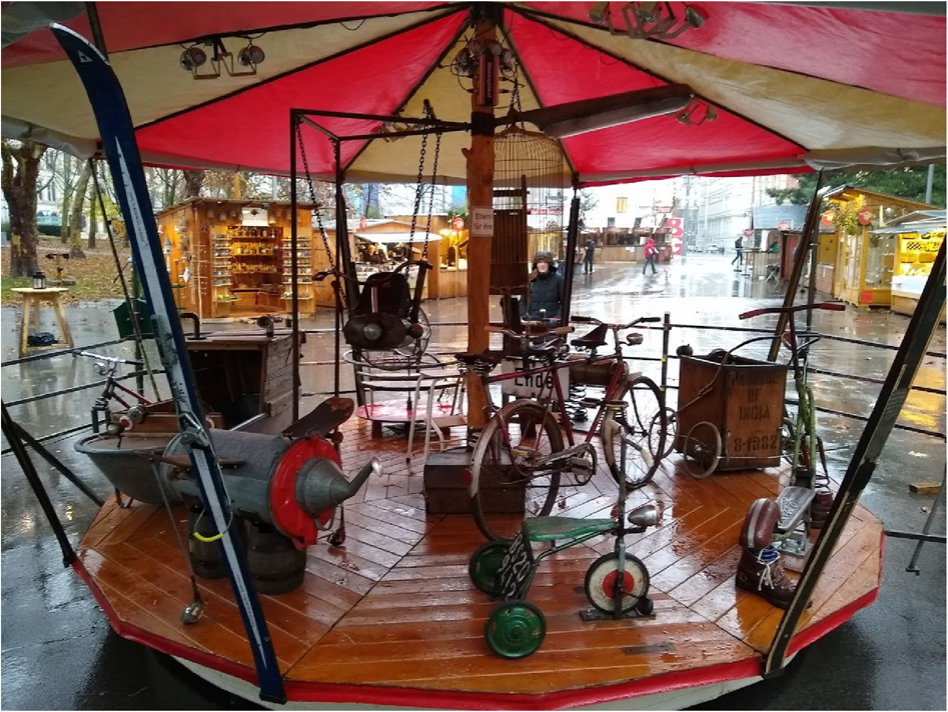
A metaphor for the circular economy in Vienna 2018: old materials generating revenue as they revolve. But energy/effort is needed to keep it moving. And even such a benign case is susceptible to rebound effects: will the revenue be reinvested in CE or in a new smartphone?

Without specific action to slow the input of virgin resources, waste reduction can reduce costs for producers, with consumers taking a share of the benefit, thereby initiating a rebound effect that results in increases in resource extraction, production and consumption, even though the quantity of waste itself may be reduced (Fig. 1). Zink and Geyer [87] have identified a set of circumstances where the environmental benefits of CE strategies are unlikely to be offset by rebound effects: these include circular products being straight substitutes for existing conventional ones (at a similar price, thereby not stimulating market growth), and such products should target satiable demand (therefore excluding electronics for the most part). Rebound effects, therefore, should be considered, but so as to maximise net overall benefit rather than to excuse inactivity.

The fact that CE is presented as a business opportunity within the current capitalist framework should arouse some suspicion. In most cases, CE is presented with the best of intentions, but it is susceptible to greenwashing and—ultimately—many products associated with CE are designed or redesigned to meet wants rather than needs, and the producer intends to make more of them, regardless of the business model. Whilst arguing for change, CE presents a vision that we can carry on living our lives as normal, with business adapting to serve every whim and an unrivalled and unverified faith in technological optimism. However, the science is clear: the challenges we are facing are enormous [68] and so we should be prepared to change rather than carry on living as normal.

As CE is typically articulated, there is no promise of simultaneous progress on the three foundations of sustainability—environment, society and economy. Each leg of the 3-legged stool is dependent on the other two to provide a secure connection between the surface we inhabit and the ‘world turtle’ on which it (mythologically) rests [88]. CE can narrow the discourse in relation to all three legs. For example, with respect to the environment, the focus on primary resources can promote a utilitarian approach to the environment, with a focus on natural capital rather than recognising biodiversity for its own sake [89]. This can be seen in how commercial forests are managed: species are chosen to maximise yield [90]. With respect to society, CBMs can provide benefit to those directly involved, but often without due consideration to people who have no stake, thereby incentivising the rentiers rather than the producers. Cutting out the middleperson can seem an attractive proposition—until we consider that we are almost all, to an extent, middlepeople.

## Where Next?

By this point, it should be clear that for such a simple phrase, ‘circular economy’ disguises a wealth of complexity and challenges which should cause us to ask how it can be useful. Arguably, the discipline is at a crucial moment where amongst its hundreds of definitions we still need to find and agree on a model that determines CE’s future fate either as the paradigm shift that the world needed in the twenty-first century or capitalism 2.0 redressed.

Circular things have more wholesome and organic connotations (the circle of life, the hydrological cycle, bicycle wheels) than linear ones (simplistic, one-dimensional) [91], so there is an immediate appeal to take the concept in uncritically. However, as a counterpoint, according to a popular search engine^1^, vicious circles are five times as common as the virtuous variety. It would be helpful to ask what conditions are needed to support a virtuous CE rather than a vicious one (for instance, a CE that meets the brief regarding economy and resources, but at the expense of human dignity, creativity and social equality).

A circular economy in which nothing is wasted can never be achieved [92], but humanity can at least move towards a circular ideal. On the other hand, we know that a modern zero-carbon economy is technically achievable by leaving fossil fuel in the ground and doing the best we can with renewable resources, for instance. Global lockdowns imposed to mitigate the spread of, and deaths caused by the COVID-19 pandemic, caused the largest drop in emissions ever recorded since records began [93]. The economy has certainly been hit hard as a result [94], emphasising how the world still resembles a see-saw with the economy at one end, and the environment at the other. If crucial tipping points have not yet been crossed, then going ‘zero-carbon’ will prevent catastrophic climate change. In fact, a zero-carbon economy—currently still a theoretical benchmark—is a basic pre-condition for a circular economy, as energy inputs will always be required to keep the circular economy turning. Therefore we should ask how CE might be a distraction from greater goals, and how it might be a key plank in the strategy for achieving such goals.

At some point in the distant future, humanity might have extracted all the mineral resources it needs to last for generations beyond. With global fertility rate collapsing, and China’s population supposed to halve by 2100 [95], will we be able to recognise when we have extracted enough to keep us going? Until that point, however, a CE continues to rely on extraction, as the requirement for new buildings and products—for instance to raise living standards in the global south to a reasonable level—continues to grow, always outstripping the availability of materials from end-of-life buildings and products. Thus, even a perfect scenario involves continuation of extraction for the foreseeable future. Perfection, in the form of zero waste, is itself a chimera [92]: assemblies wear out; materials degrade under environmental influences such as ultraviolet radiation and acidic rain; they erode, leaving micro-particles in water (from clothes washing), on surfaces (vehicle tyres), in the air (combustion); and even polymers once assumed to have been indigestible, such as PET, have been found to be susceptible to bacteriological degradation, potentially into its constituent monomers thereby offering a route towards a closed-loop recycling system [96].

Imagine a zero-carbon future: yes, sharing of resources and making the best use of what we have are likely to be part of it, but the main features of this future are likely to be either reverting to a ‘simpler’ way of life (appealing to a minority), or betting on technological progress to get us there. Various combinations of wind, solar, nuclear, energy storage and carbon capture would do the heavy lifting, enabled by data services, and maintain a recognisable standard of living. It is however important not to buy into unrealistic techno-optimism [97].

Now imagine a circular future. What does this look like? Perhaps a world in which objects are built to last indefinitely (or, better, exactly as long as needed, to avoid over-engineering), but made with minimal resource inputs: this would however be a world in which the needs of future generations are known to the present. Or a world without single-use plastics: but how do we replace or displace them? Single-use non-plastics, with their own challenges? A reverse logistics infrastructure for reusable packaging? Food products in their new genetically modified skins, enhanced for resilience?

A circular future is a less clear goal than the zero-carbon future, and therefore more difficult to model and plot a path towards it. Thus we should see circular economy as a journey, not a destination, which we embrace along with its contradictions. This should be within a sustainability context, and if weaker visions and definitions of CE do not become extinct, then it should be understood as belonging firmly to the economic pillar. In that case, CE will have to work alongside (and sometimes in opposition to) alternative visions for the environment and society, and their associated tools and metrics. In other words, we should jump on to the bandwagon, but should also upgrade the brakes and steering.

## Data Availability

Not applicable.

## Footnotes

1 72 million results from a Google search for the term ‘vicious circle’, and 14.3 million for ‘virtuous circle’ Nov 2020.

## Abbreviations

BAMB: building as material banks
BIM: building information modelling
CBM: circular business models
CE: circular economy
EMF: Ellen MacArthur Foundation
EU: European Union
GHG: greenhouse gases
PET: polyethylene terephthalate
